# Factors associated with bleeding events during catheter ablation with uninterrupted periprocedural edoxaban for atrial fibrillation: a subanalysis of the KYU-RABLE study

**DOI:** 10.1186/s12959-021-00305-7

**Published:** 2021-08-03

**Authors:** Ichitaro Abe, Naohiko Takahashi, Yasushi Mukai, Tetsuya Kimura, Keita Yamaguchi, Atsushi Takita, Hideki Origasa, Ken Okumura, on behalf of the KYU-RABLE Investigators

**Affiliations:** 1 Department of Cardiology and Clinical Examination, Faculty of Medicine, Oita University, 1-1 Idaigaoka, Hasame-machi, Yufu, Oita 879-5593 Japan; 2 Cardiology Division, Japanese Red Cross Fukuoka Hospital, 3-1-1 Ogusu, Minami-ku, Fukuoka, 815-8555 Japan; 3 Medical Science Department, Daiichi Sankyo Co., Ltd., 3-5-1 Nihombashihoncho, Chuo-ku, Tokyo, 103-8426 Japan; 4 Data Intelligence Department, Daiichi Sankyo Co., Ltd., 3-5-1 Nihombashihoncho, Chuo-ku, Tokyo, 103-8426 Japan; 5 Professor emeritus, The University of Toyama, 2630 Sugitani, Toyama, 930-0194 Japan; 6 Division of Cardiology, Saiseikai Kumamoto Hospital Cardiovascular Center, 5-3-1 Chikami, Minami Ward, Kumamoto, 861-4193 Japan

**Keywords:** Bleeding, Catheter ablation, Edoxaban

## Abstract

**Background:**

Data are limited on patient background characteristics associated with catheter ablation (CA)-related bleeding events in Japanese patients with non-valvular atrial fibrillation receiving uninterrupted periprocedural edoxaban. This subanalysis of the KYU-RABLE study focused on univariate and multivariate analyses to identify correlations between bleeding events and baseline patient demographics and CA-related characteristics.

**Methods:**

Patients with non-valvular atrial fibrillation (NVAF) enrolled from the KYU-RABLE study were included in the study. We performed univariate and multivariate analyses to investigate the correlation of major, minor, and clinically relevant non-major bleeding events with the patient baseline data at enrollment, and with CA procedures.

**Results:**

A total of 513 NVAF patients were included in the full analysis set. Univariate analysis showed that the incidence of the bleeding events was higher in patients with HAS-BLED score ≥ 3 compared with those with a score < 3 (odds ratio [OR]: 9.48, 95% CI: 2.36–38.01; *p* = 0.002), in those with creatinine clearance (CrCL) ≤50 mL/min compared with those with CrCL > 50 mL/min (OR: 10.59, 95% CI: 3.65–30.79; *p* < 0.0001), and in those receiving edoxaban 30 mg compared with those receiving edoxaban 60 mg (OR: 3.49, 95% CI: 1.18–10.38; *p* = 0.025). Multivariate analysis showed that HAS-BLED score ≥ 3 (OR: 7.93, 95% CI: 1.66–37.88; *p* = 0.0094) and CrCl ≤ 50 mL/min (OR: 7.78, 95% CI: 2.17–27.90; *p* = 0.0016) were significant predictors of bleeding events among KYU-RABLE patients.

**Conclusions:**

These predictors of CA-related bleeding events may allow informed decision-making and better AF patient selection for CA with uninterrupted periprocedural edoxaban.

**Trial registration:**

KYU-RABLE, UMIN000029693. Registered 1 December 2017.

## Background

Atrial fibrillation (AF) is the most common cardiac arrhythmia, especially in elderly populations [1]. Among AF treatments, catheter ablation (CA) has been developed in the past decades, and there is increasing evidence on its efficacy and safety versus antiarrhythmic drugs [2, 3]. However, several challenges remain with CA, such as AF recurrence and the occurrence of severe CA-related complications (i.e., bleeding, stroke, and esophageal damage) [2]. Some studies have analyzed factors that could potentially relate to the occurrence of CA-related adverse events (AEs), including age, sex, body weight, antiplatelet drug use, initial ablation session, and chronic kidney disease [4–7]. Nevertheless, limited data are available on the association between patient background characteristics and bleeding events in Japanese patients.

The efficacy and safety of uninterrupted direct oral anticoagulants (DOACs) during the CA periprocedural period have been previously reported in the KYU-RABLE [8] and ELIMINATE-AF [9] studies. The main objective of this subanalysis of the KYU-RABLE study was to clarify the potential predictors of bleeding events during and after CA among Japanese nonvalvular AF (NVAF) patients receiving uninterrupted periprocedural edoxaban.

## Methods

### Study design and patients

The details of the KYU-RABLE study, including the ethical considerations of the study, have been published previously [8]. Briefly, the KYU-RABLE study was a prospective, multicenter, single-arm interventional study conducted between 1 December 2017 and 21 September 2018 at 23 institutions in Japan. Eligible patients were those aged ≥20 years who were scheduled to undergo CA for NVAF. Patients were excluded if they had any contraindications for edoxaban treatment or any contraindications for CA, a creatinine clearance (CrCL) < 30 mL/min, history of thromboembolism or myocardial infarction within 2 months before enrollment, history of intracranial, intraocular, spinal, retroperitoneal, or atraumatic intra-articular bleeding, or history of gastrointestinal bleeding or major bleeding per the International Society on Thrombosis and Haemostasis [ISTH] definition within 4 weeks before enrollment.

The study treatment consisted of edoxaban 60 mg administered once daily in the morning for ≥4 weeks before CA and continued for 4 weeks ±7 days after CA. On the day of CA, edoxaban was administered not before but after CA (i.e., one dose delayed administration). A reduced dose of 30 mg was given if patients met the dose adjustment criteria [8]. Investigators at each site selected the type of CA procedure, energy source, and the use of heparin and dosage for maintenance of activated clotting time (ACT) > 300 s during the procedure.

### Study outcomes and measures

Data related to patient baseline demographic and background clinical characteristics were collected at enrollment. Details of the CA, CA-related complications, periprocedural treatment, clinical events, and study endpoints were collected during the study period. Edoxaban plasma concentration was measured 1 h before CA and on the day after CA. Bleeding events were collected during the CA procedure period up to the end of the study.

Major bleeding was defined according to the ISTH definition (fatal bleeding; retroperitoneal, intracranial, intraocular, intrathecal, intra-articular, or pericardial bleeding; intramuscular bleeding with symptoms of compartment syndrome; or clinically overt bleeding that required a transfusion). Clinically relevant non-major bleeding was defined as clinically overt bleeding requiring intervention, including laboratory tests, diagnostic imaging, endoscopy, colonoscopy, cystoscopy, bronchoscopy, and compression hemostasis. Minor (not clinically relevant) bleeding was defined as evident bleeding that did not meet the criteria for major bleeding or clinically relevant non-major bleeding.

### Statistical analysis

Clinical events were analyzed in the full analysis set. Odds ratios and 95% confidence intervals (CIs) were calculated using a logistic regression model. For the present subanalysis, univariate and multivariate analyses were performed to identify associations between major, minor, and clinically relevant non-major bleeding events with baseline patient demographics and clinical characteristics, including age, sex, AF type, CHADS_2_ and HAS-BLED scores, renal function by CrCL, CA-related factors, including the type of CA (radiofrequency ablation or cryoballoon ablation), the dose of perioperative heparin, edoxaban dose and plasma concentration of edoxaban 1 h before CA, time from CA termination to restart of edoxaban administration on the CA day, and maximum ACT during CA. Factors included in the final multivariate model were selected based on a two-tailed significance level of 5% as these factors significantly influenced the incidence of events in the univariate model. All statistical analyses were performed using SAS version 9.4 (SAS Institute Inc., Cary, NC, USA).

## Results

### Patient disposition and background characteristics and CA procedure details

A total of 513 NVAF patients were included in the full analysis set. The baseline and demographic characteristics and the details related to the CA procedure have been described previously [8].

### Bleeding events

There were 18 bleeding events in 16 patients (3.1%). Of these, there was one major bleeding event (cardiac tamponade). Seven clinically relevant non-major bleeding events occurred, mainly at the venous puncture site. There were eight cases of minor bleeding events, primarily consisting of puncture site hematoma at the groin. Two patients presented two events of groin hematoma, and the first event was considered for the analyses. Most bleeding events occurred during the same day of the CA and from the day of the CA to day 7 after the CA. No bleeding events occurred between days 8 and 35 of follow-up.

### Univariate analysis

The results in the univariate analysis are shown in Table 1. Regarding patient background factors, the incidence of the bleeding events was higher in patients with HAS-BLED score ≥ 3 compared with those with a score < 3 (odds ratio [OR]: 9.48, 95% CI: 2.36–38.01; *p* = 0.002), and in those with CrCL ≤50 mL/min compared with those with CrCL > 50 mL/min (OR: 10.59, 95% CI: 3.65–30.79; *p* < 0.0001). Sex, age, AF type, CA type, and CHADS_2_ score were not correlated with bleeding events. Regarding the factors related to the CA procedure, the incidence was higher in patients receiving edoxaban 30 mg compared with those receiving edoxaban 60 mg (OR: 3.49, 95% CI: 1.18–10.38; *p* = 0.025). The heparin dose, the plasma concentration of edoxaban, time from CA termination to restart of edoxaban administration, and ACT were not correlated with bleeding events.

**Table 1:** Univariate analysis of factors associated with bleeding events during CA with uninterrupted periprocedural edoxaban

Factors	Univariate OR (95% CI)	*p*-value
Sex		
Female vs male	1.182 (0.397–3.519)	0.7633
Age, years		
65–74 vs < 65	2.604 (0.682–9.949)	0.1617
≥ 75 vs < 65	3.835 (0.839–17.542)	0.0831
AF type		
Non-paroxysmal vs paroxysmal	2.144 (0.752–6.116)	0.1539
CA surgical type		
Radiofrequency vs cryoballoon	1.948 (0.433–8.765)	0.3848
CHADS_2_ score		
≥ 2 vs < 2	2.194 (0.781–6.162)	0.1360
HAS-BLED score		
≥ 3 vs < 3	9.478 (2.363–38.014)	0.0015
Renal function (CrCL), mL/min		
≤ 50 vs > 50	10.594 (3.645–30.790)	< 0.0001
Edoxaban dose the day before CA, mg		
30 vs 60	3.492 (1.175–10.378)	0.0245
Time from CA termination to restart of edoxaban administration on the day of CA, hours		
≥ 3 and < 6 vs < 3	1.159 (0.127–10.570)	0.8956
≥ 6 vs < 3	2.124 (0.261–17.314)	0.4817
Plasma concentration of edoxaban before CA (ng/mL)	1.005 (0.974–1.038)	0.7331
Perioperative heparin dose (unit)	1.003 (0.993–1.013)	0.5387
Maximum ACT during CA (s)	1.011 (0.951–1.075)	0.7222

For each factor, the category to the right is the reference. The final 3 items were considered continuous variables. Factors found to be significant in the univariate analysis were subsequently entered into multivariate analysis

*ACT* activated clotting time; *AF* atrial fibrillation; *CA* catheter ablation; *CI* confidence interval; *CrCL* creatinine clearance; *OR* odds ratio

### Multivariate analysis

**Fig. 1:**
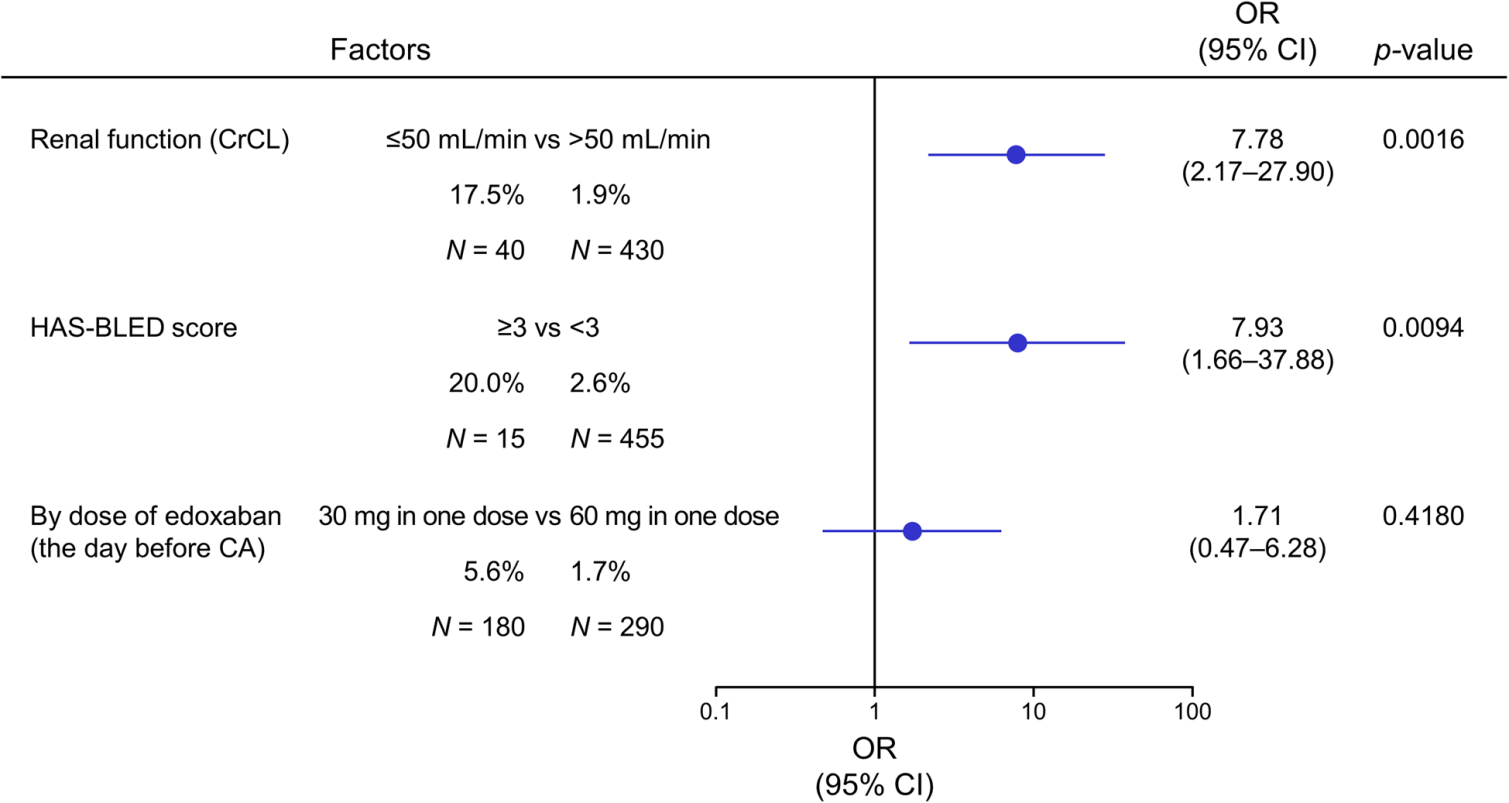
Forest plot of multivariate analysis of the significant factors identified in the univariate analysis. CA, catheter ablation; CI, confidence interval; CrCL, creatinine clearance; OR, odds ratio

Significant factors in the univariate analysis were then included in the multivariate analysis (Fig. 1). HAS-BLED score ≥ 3 (OR: 7.93, 95% CI: 1.66–37.88; *p* = 0.0094) and decreased renal function (CrCL ≤50 mL/min) (OR: 7.78, 95% CI: 2.17–27.90; *p* = 0.0016) were identified as significant predictors of bleeding events. Edoxaban at a dose of 30 mg versus a dose of 60 mg was not a significant predictor.

## Discussion

This subanalysis of the KYU-RABLE study [8] aimed to identify possible factors among patient backgrounds and CA-related characteristics that could predict the bleeding events among Japanese patients with NVAF undergoing CA and receiving uninterrupted periprocedural anticoagulation with edoxaban. Patients with HAS-BLED score ≥ 3 and decreased renal function (CrCL ≤50 mL/min) had a higher incidence of bleeding events.

The KYU-RABLE study is unique because the once-daily DOAC, edoxaban, was administered in the morning during the periprocedural period as well as on the day of CA. However, the key feature of this study is that edoxaban was administered after the procedure once hemostasis was confirmed rather than in the morning before the CA. Thus, rather than interrupting edoxaban administration on the day of CA, edoxaban administration was delayed until after the procedure, namely uninterrupted edoxaban with one dose delayed administration. Two randomized studies (VENTURE-AF and ELIMINATE-AF) [9, 10] compared the effectiveness and safety of once-daily DOACs (rivaroxaban and edoxaban) versus uninterrupted warfarin and reported similar bleeding event rates between the 2 groups. In these studies, once-daily DOACs were administered in the evening on the day of the CA rather than in the morning. Therefore, these once-daily DOACs were not administered before but after the procedure, as per the protocol of uninterrupted periprocedural DOAC with one dose delayed administration. Thus, in these studies [9, 10], the CA procedure was done within 24 h after the last DOAC dose. The package inserts of once-daily DOACs in Japan state that it is desirable to perform invasive procedures with an interval greater than 24 h after the last dose to decrease bleeding risk. The present protocol of edoxaban administration was consistent with the recommendation in the package insert and the DOAC dosing used widely in Japan in patients undergoing CA [11].

In this analysis, no thromboembolism occurred when the CA procedure was performed mostly within an interval greater than 24 h after the last edoxaban administration [8]. Only one major bleeding event of cardiac tamponade was observed, which was considered procedure-related, and it was managed with a pericardiocentesis. However, approximately 3% of the patients presented bleeding complications, which resulted in 18 events in 16 patients, mostly on the day of the CA, and some within the following week. All of these bleeding events were classed as clinically relevant non-major bleeding events (7 events) and minor bleeding events (10 events). Because edoxaban was administered not before but after the procedure on the day of CA to reduce the bleeding risk, it is vital to identify the predictors of bleeding events just after the procedure.

In the univariate analysis, decreased renal function, HAS-BLED score ≥ 3, and reduced edoxaban dose (30 mg) were significantly associated with bleeding events. After correction for patient background factors in the multivariate analysis, decreased renal function and HAS-BLED score ≥ 3 remained significant. The association between the 30-mg edoxaban dose and bleeding events was likely explained by the dose reduction criteria, such as reduced renal function and low body weight. These findings were aligned with the results of a recent subanalysis of the Japanese Anticoagulation Regimen Exploration in AF Catheter Ablation Registries that demonstrated HAS-BLED score ≥ 3 as a predictor of bleeding complications [12]. A previous Japanese study evaluated periprocedural uninterrupted DOAC compared with uninterrupted warfarin in patients undergoing CA for AF stratified by various renal function groups. It showed a higher risk of periprocedural bleeding events in patients with chronic kidney disease [6], which is consistent with our findings.

Among elderly patients with AF undergoing CA with uninterrupted DOACs or warfarin, lower body weight (OR: 0.96; *p* = 0.010) and antiplatelet drug use (OR: 2.21; *p* = 0.039) were reported as independent predictors of CA-related AEs [5]. Another study found that a higher CHA_2_DS_2_-VASc score and early institutional experience were independent predictors of CA-related AEs [7]. Female sex was associated with a greater risk of complications among patients with AF undergoing left atrial CA with continuous periprocedural rivaroxaban (OR: 1.96; 95% CI: 1.10–3.49) [4]. Notably, sex, age, AF type, CA type, CHADS_2_ score, heparin dose, plasma concentration of edoxaban, time from CA termination to the restart of edoxaban administration, and ACT were not correlated with bleeding events during or after CA in the present subanalysis. The differences in the results may be attributable to the differences in the protocols of oral anticoagulant administration across studies.

The main limitations of the present analysis included the open-label design, lack of comparator, and short follow-up period. Further, this study was not originally designed to identify factors associated with bleeding events during CA with uninterrupted periprocedural edoxaban for AF. Finally, this was an exploratory study, and it was difficult to analyze several factors due to the scarcity of events.

## Conclusions

In this subanalysis of the KYU-RABLE study, the multivariate analysis indicated that HAS-BLED score ≥ 3 and decreased renal function (CrCL ≤50 mL/min) were associated with increased bleeding events. It is important to identify potential predictors of CA-related AEs, as this allows for informed decision-making and better patient selection that may derive improved outcomes and other benefits for AF patients undergoing CA with uninterrupted periprocedural DOAC.

## Data Availability

The deidentified participant data will be shared on a request basis and it consists of the deidentified data that support the results presented in this article, as well as the study protocol. Please directly contact the corresponding author (takanao@oita-u.ac.jp) to request data sharing. The data will become available from submission up to 36 months after article publication. For data sharing, the researchers should provide a methodologically sound proposal, which may be reviewed by a committee led by Daiichi-Sankyo. The data shared could be used for any purpose and the proposals should be directed to the corresponding author. To gain access to data sharing, data requestors will need to sign an access data agreement.

## Abbreviations

ACT: Activated clotting time
AF: Atrial fibrillation
CA: Catheter ablation
CI: Confidence interval
CrCL: Creatinine clearance
DOACs: Direct oral anticoagulants
ISTH: International Society on Thrombosis and Haemostasis
NVAF: Nonvalvular atrial fibrillation
OR: Odds ratio
